# Antibiotics that affect translation can antagonize phage infectivity by interfering with the deployment of counter-defenses

**DOI:** 10.1073/pnas.2216084120

**Published:** 2023-01-20

**Authors:** Benoit J. Pons, Tatiana Dimitriu, Edze R. Westra, Stineke van Houte

**Affiliations:** ^a^Environment and Sustainability Institute, Biosciences, University of Exeter, Penryn TR10 9FE, UK

**Keywords:** bacteriophage, CRISPR-Cas, anti-CRISPR, translation inhibitors, antibiotics

## Abstract

It is becoming increasingly clear that antibiotics can both positively and negatively impact the infectivity of bacteriophages (phage), but the underlying mechanisms often remain unclear. Here we demonstrate that antibiotics that target the protein translation machinery can fundamentally alter the outcome of bacteria–phage interactions by interfering with the production of phage-encoded counter-defense proteins. Specifically, using Pseudomonas aeruginosa PA14 and phage DMS3vir as a model, we show that bacteria with Clustered Regularly Interspaced Short Palindromic Repeat, CRISPR associated (CRISPR-Cas) immune systems have elevated levels of immunity against phage that encode anti-CRISPR (acr) genes when translation inhibitors are present in the environment. CRISPR-Cas are highly prevalent defense systems that enable bacteria to detect and destroy phage genomes in a sequence-specific manner. In response, many phages encode *acr* genes that are expressed immediately following the infection to inhibit key steps of the CRISPR-Cas immune response. Our data show that while phage-carrying *acr* genes can amplify efficiently on bacteria with CRISPR-Cas immune systems in the absence of antibiotics, the presence of antibiotics that act on protein translation prevents phage amplification, while protecting bacteria from lysis.

## Significance

Due to the current antibiotic crisis, there is a rising interest in combined phage-antibiotics therapy. Our results help to understand how antibiotics-phage synergy and antagonism depend on the molecular interactions that define phage infectivity and host immunity. Phages are thought to play a prominent role in shaping microbiota and inducing dysbiosis. Hence, insights on how antibiotics impact phage–bacteria interactions are of applied significance for animal and human health.

In natural environments, phages are estimated to be 10 times more abundant than bacteria, and to cause the lysis of 20 to 40 % of the bacterial biomass each day (1). To resist against phage infection, bacteria have evolved a wide range of defense mechanisms (2–4) organized in several “lines of defense,” providing immunity against a wide variety of viruses (5, 6). Among these defense mechanisms, the Clustered Regularly Interspaced Short Palindromic Repeat, CRISPR associated (CRISPR-Cas) system provides acquired immunity against previously encountered phages and other mobile genetic elements (7–10). It relies on the acquisition of fragments from invading phage genetic material (spacers) from earlier failed infections, which are inserted in a specific CRISPR locus on the bacterial chromosome. CRISPR RNAs (crRNAs) are then transcribed and processed from the CRISPR loci and form a surveillance complex with Cas protein(s). Guided by the crRNA, this surveillance complex recognizes sequences matching the spacer (protospacers) in invading genetic material, leading to sequence-specific cleavage of the invader. Modulation of CRISPR-Cas activity can occur through the expression of either the CRISPR array or the Cas genes. CRISPR-Cas expression varies across bacterial and archaeal species and can either be constitutive or can be activated in response to specific stimuli, such as cell density, phage contact, or genetic material detection (11). CRISPR-Cas systems are found in approximately 40% of bacterial genomes, making them one of the most prevalent defense systems identified so far, and are classified into two classes, six types and 33 subtypes based on differences in the number and nature of associated proteins (10).

Phages are not defenseless against CRISPR-Cas systems as they have evolved counter-defense mechanisms during their struggle against their bacterial foes (3, 12). Some phages encode small peptides, called anti-CRISPR proteins (Acr), that hinder the binding or cleavage of the phage genome by the CRISPR-Cas system (13–15). Acrs are not a priori present in the phage particles but are expressed only at the start of the infection, at very high levels (16, 17). When faced with a CRISPR-immune host (i.e., that has a fully matching spacer targeting the phage), Acr production is not always fast enough to completely inactivate the surveillance machinery, leading to cleavage of the phage genetic material (18, 19). However, despite phage cleavage, the Acr protein produced will leave bacteria in an immunosuppressed state, in which part of the surveillance complexes are inhibited by Acr, thus increasing the probability that a subsequently infecting phage can successfully replicate on this immunosuppressed host (18–20).

Recent work has shown that the presence of bacteriostatic antibiotics (antibiotics inhibiting cell growth without killing) favor the acquisition of CRISPR-Cas immunity during infection with phages that lack Acr activity (21). Sub-inhibitory doses of bacteriostatic antibiotics slow down both bacterial growth and phage replication, thereby lengthening the phage replication cycle and hence allowing more time for bacteria to acquire new spacers against the phage before being lysed. Phage-antibiotic interactions can range from synergy (22, 23) to antagonism (24). While often a mechanistic explanation for the observed interaction is lacking (25) and references herein), several different mechanisms have been identified, including antagonism caused by a decreased host RNA synthesis (26, 27) and synergy mediated by phage-mediated impairment of antibiotic resistance development coupled by an antibiotic-mediated hindrance of phage resistance apparition (28). The paper by Dimitriu et al. (21) identifies a previously unknown mechanism driving antagonism between bacteriostatic antibiotics and phages that infect bacteria carrying a functional CRISPR-Cas system.

Antibiotics impair bacterial growth or kill cells by acting on various molecular targets (29). One of the most common targets is the ribosome, an enzymatic complex responsible for the translation of messenger RNA into functional polypeptides, and hence essential for bacterial survival and growth. Antibiotic compounds from a wide variety of classes bind to only a few sites in the 30S or 50S ribosomal subunits, which disturbs initiation, elongation, or termination of translation, even at sub-inhibitory antibiotic doses (30–33).

Since successful Acr-phage amplification relies on the strong production of Acrs at the onset of infection, we hypothesized that translation inhibitor antibiotics have the potential to interfere with Acr-induced immunosuppression, thereby effectively re-sensitizing the phage to full CRISPR-Cas immunity. Using the *Pseudomonas aeruginosa* strain PA14 and its lytic phage DMS3*vir* as a model system, we show that subinhibitory doses of translation inhibitory antibiotics block Acr-induced immunosuppression and hence successful phage replication. Concomitantly, infected bacteria benefit from the presence of these antibiotics, suggesting an antagonistic interaction between translation inhibitor antibiotics and Acr-phages infecting CRISPR-immune bacteria.

## Results

### Translation Inhibitors Antibiotics Disrupt Acr-Mediated Inhibition of CRISPR-Cas Immunity.

**Fig. 1.**
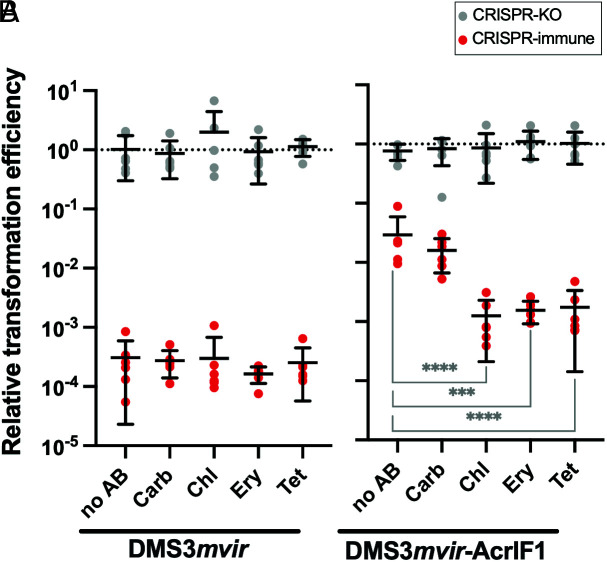
Bacteriostatic translation inhibitors disrupt Acr-mediated inhibition of the CRISPR system. Relative transformation efficiencies (targeted plasmid/non-targeted plasmid) of PA14 CRISPR-KO (grey data points) or CRISPR-immune (red data points) pre-infected with phage DMS3*mvir* (*A*) or DMS3*mvir*-AcrIF1 (*B*), in the absence (no AB) or presence of different antibiotics (*SI Appendix,* Table S1). Each data point represents an independent biological replicate (n = 6), and the mean ± SD for each treatment is displayed as black bars. Asterisks show treatments that are different from the no-antibiotic control (Dunnett, ***0.0001 < *P* < 0.001, ****P < 0.0001).

The strong and early production of Acrs (16, 17) during phage infection suggests that their efficiency might be impaired in the presence of translation inhibitor antibiotics. To test the hypothesis that these antibiotics may impact the effectiveness of Acr proteins during phage infection, we infected *P. aeruginosa* UCBPP-PA14 (PA14) with the lytic phage DMS3*mvir*-AcrIF1, which carries a type I-F *acr* gene (34), or the isogenic control phage DMS3*mvir*, which does not carry a type I-F *acr*. Wild-type bacteria, which carry a type I-F CRISPR-Cas immune system that targets phage DMS*mvir* and DMS3*mvir*-AcrIF1 a priori (CRISPR-immune), or an isogenic CRISPR-knockout (CRISPR-KO) control strain were infected at low multiplicity of infection (MOI) with either DMS3*mvir* or DMS3*mvir*-AcrIF1. Bacteria were grown for 2 h in the presence of phage and one of four antibiotics belonging to different chemical classes (*SI Appendix*, Table S1). Three of these antibiotics (chloramphenicol, Chl, erythromycin, Ery and tetracycline, Tet) target protein translation, (21, 30), while one (carbenicillin, Carb) acts on cell wall synthesis and is not expected to have a direct effect on translation (35). The antibiotics were used at minimum inhibitory concentration (MIC) for Ery and sub-MIC doses for the other antibiotics: 0.1 × MIC, 0.8 × MIC and 0.25 × MIC for Carb, Chl, and Tet, respectively. At the selected doses LacZ protein production was disturbed in the presence of Chl, Ery, and Tet, but not by Carb (*SI Appendix,* Fig. S1). After washing away antibiotics and phage, we assessed the relative transformation efficiency (RTE) of the bacteria by transforming them with a plasmid either targeted (T) or not targeted (NT) by the PA14 CRISPR-Cas system (Fig. 1).

Neither the phage treatment nor the antibiotic treatment had influence on the RTE of the CRISPR-KO strain (Fig. 1). As previously shown (19), the CRISPR-immune strain infected with DMS3*mvir*-AcrIF1 displayed a higher RTE than when infected with DMS3*mvir* due to lasting immunosuppression by the Acr. None of the antibiotic treatments affected the RTE of the CRISPR-immune strain infected with DMS3*mvir* compared to the no-antibiotic control (*P *> 0.9 for all antibiotics) (Fig. 1*A*). In contrast, treatment with Chl, Ery, and Tet significantly decreased the RTE when the CRISPR-immune strain was infected with DMS3*mvir*-AcrIF1 compared to the no-antibiotic control (*P* < 0.0001, *P* = 0.0004, *P* < 0.0001, respectively; Fig. 1*B*). Conversely, Carb did not affect immunosuppression in CRISPR-immune cells by DMS3*mvir*-AcrIF1 (*P* = 0.80). Overall, these results suggest that Chl, Ery, and Tet, but not Carb can block immunosuppression induced by AcrIF1.

### Translation Inhibitor Antibiotics Decrease Infection Efficiency of Acr-Phage.

**Fig. 2.**
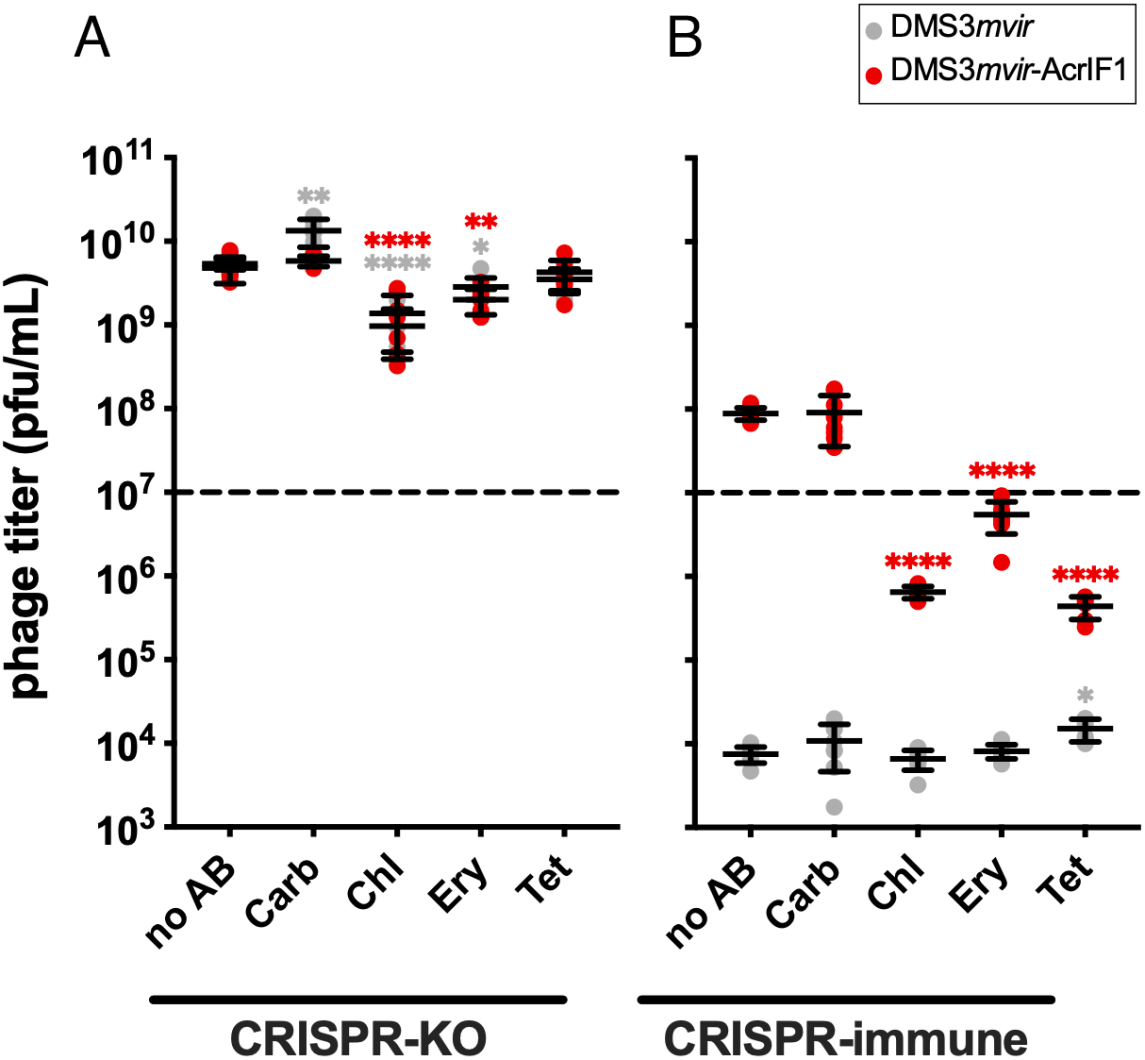
Translation inhibitor antibiotics decrease infection efficiency of Acr phage. Effects of different antibiotic treatments (*SI Appendix,* Table S1) on DMS3*mvir* (grey data points) or DMS3*mvir*-AcrIF1 (red data points) titer after 24 h of infection on PA14 CRISPR-KO (*A*) or CRISPR immune cells (*B*). The dashed line indicates the phage titer at t = 0 h. Each data point represents an independent biological replicate (n = 8) with limit of detection at 250 plaque-forming units (PFUs)/mL, and the mean ± SD for each treatment is displayed as black bars. Asterisks show treatments that are different from the no-antibiotic control (Dunnett, *0.01 < *P* < 0.05, **0.001 < *P *<0.01, ***0.0001 < *P* <0.001, ****P <0.0001).

Based on the observation that some translation inhibitors interfere with Acr-induced immunosuppression, we then hypothesized that these antibiotics would affect DMS3*mvir*-AcrIF1 replication in a CRISPR-immune host. To test this, we infected CRISPR-KO and CRISPR-immune cells with DMS3*mvir* or DMS3*mvir*-AcrIF1 at MOI = 1 in the presence or absence of antibiotics and measured phage titers after 24 h (Fig. 2). Antibiotics were used with the same inhibitory or subinhibitory concentration as in the RTE experiment. Control experiments with a CRISPR-KO strain showed that antibiotics have a moderate impact on phage amplification (Fig. 2*A*). Crucially, there was no difference between phage DMS3*mvir* and DMS3*mvir*-AcrIF1. In contrast, in CRISPR-immune bacteria, phages were only able to amplify the above input titer when carrying the *acrIF1* gene (Fig. 2*B*). Moreover, in the presence of any of the three translation inhibitors, DMS3*mvir*-AcrIF1 was unable to amplify (*P* < 0.0001 for all treatments, compared with the no-antibiotic control), whereas Carb did not interfere with phage amplification (*P* > 0.99)(24, 36, 37). Thus, these results show that the three translation inhibitors Chl, Ery, and Tet interfere with the ability of DMS3*mvir*-AcrIF1 to block CRISPR-Cas immunity.

### Translation Inhibitor Antibiotics Protect CRISPR-Immune Cells from Acr-Phages.

**Fig. 3.**
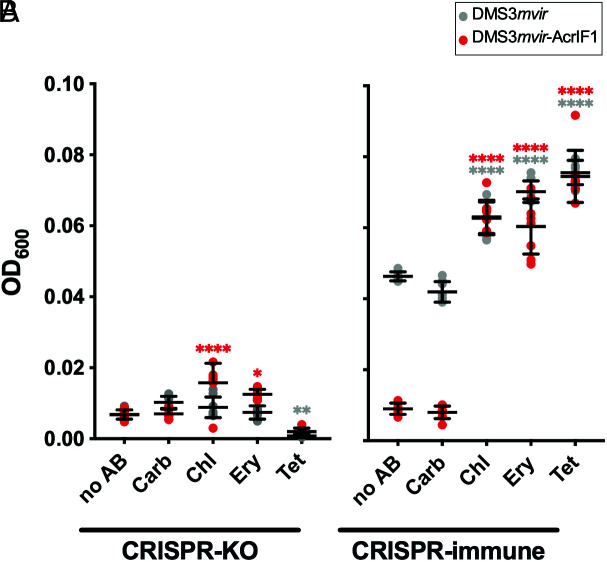
Translation inhibitor antibiotics protect CRISPR-immune cells from Acr-mediated lysis. Effects of the different antibiotic treatments (*SI Appendix,* Table S1) on bacterial OD_600_ of PA14 CRISPR-KO (*A*) or CRISPR-immune (*B*) after 24 h of infection by DMS3mvir (grey data points) or DMS3mvir-AcrIF1 (red data points). Each data point represents an independent biological replicate (n = 8), and the mean ± SD for each treatment is displayed as black bars. Asterisks show treatments that are different from the no-antibiotic control (Dunnett, *0.01 < *P* < 0.05, **0.001 < *P *<0.01, ****P < 0.0001).

Since Chl, Ery, and Tet hinder DMS3*mvir*-AcrIF1 amplification on CRISPR-immune bacteria, we predicted that these antibiotics would protect infected cells from phage-induced lysis. We thus evaluated the impact of antibiotics on the optical density at l = 600 nm (OD_600_) of PA14 CRISPR-KO or CRISPR-immune after 24 h of infection by either DMS3*mvir* or DMS3*mvir*-AcrIF1 at MOI = 1 (Fig. 3). As expected, the presence of phage prevented the CRISPR-KO growth compared to cells growing without phage (Fig. 3 and *SI Appendix,* Fig. S2). Moreover, for each antibiotic treatment, bacterial growth of CRISPR-KO cells following phage infection was independent of the presence or absence of phage-carrying *acrIF1* (Fig. 3*A*). Conversely, in the absence of antibiotics, the OD_600_ of CRISPR-immune cells was higher following infection with DMS3*mvir* than DMS3*mvir*-AcrIF1, and the presence of Carb did not affect this pattern (Fig. 3*B*). However, the presence of Chl, Ery, and Tet allowed CRISPR-immune cells to grow to similar OD_600_ when infected by DMS3*mvir* and DMS3*mvir*-AcrIF1, thus removing any effect of phage-encoded *acrIF1* on host growth. Overall, these results suggest that Chl, Ery, and Tet increase the ability of CRISPR-immune bacteria to resist lysis by Acr-phage, causing phage-antibiotics antagonism in those instances.

### Gentamycin Prevents Phage Replication in a CRISPR- and Acr-Independent Manner.

We also selected and tested a fourth translation inhibitor antibiotic, gentamycin (Gm) (*SI Appendix,* Table S1). While exposure to Gm at 0.5 × MIC efficiently prevents protein expression in a CRISPR-KO strain (*SI Appendix,* Fig. S3*A*), Gm was unable to affect the RTE of the CRISPR-immune strain (*SI Appendix,* Fig. S3*B*). Gm was then tested for its ability to disturb phage infection and bacterial growth. Gm prevented both DMS3mvir and DMS3mvir-AcrIF1 from amplifying, both on CRISPR-KO and CRISPR-immune hosts (*SI Appendix,* Fig. S3*C*). This suggests that Gm causes an overall phage fitness decrease, in line with previous studies showing that aminoglycosides can inhibit phage infectivity (24, 36, 37). Moreover, treatment with Gm resulted in levels of bacterial growth similar to the no-phage controls, independently from the presence of a functional CRISPR-Cas system in the host or the *acrIF1* gene in the phage (*SI Appendix,* Fig. S3*D*). This further supports a direct effect of Gm on phage infectivity.

## Discussion

In Mu-like phages, such as DMS3, *acr* genes are expressed before genes classified as early expressed, including the transposase (16, 38). Evidence that some Acr-carrying phages need several infections of the same cell to successfully overcome CRISPR-mediated immunity (18, 19) suggests that Acr protein levels, and thus its production, are critical to its ability to overcome CRISPR-Cas immunity. We consequently hypothesized that disturbance in Acr production might affect the outcome of Acr-phage infection on a CRISPR-immune host. More specifically, we propose that antibiotics inhibiting protein translation disturb Acr production and thus interfere with the effectiveness of Acr-phages infecting CRISPR-immune bacteria.

We show here that Acr activity is indeed reduced when CRISPR-immune cells were co-exposed to Acr-phages and the translation inhibiting antibiotics Chl, Ery, or Tet (Fig. 1). Exposure to these antibiotics significantly reduced Acr-phage titer (Fig. 2) and allowed CRISPR-immune bacteria to grow to substantially higher density than without antibiotics (Fig. 3). Consistent with our hypothesis, these translation inhibitor antibiotics hindered Acr-mediated immunosuppression, presumably through their ability to disturb protein production, even at sub-MIC doses (39–41). This led to disturbed phage replication, thus providing protection to CRISPR-immune bacteria against Acr-phages.

Despite having no effect on Acr immunosuppression activity, Gm had a similar impact on phage titer and bacterial growth than the previous three antibiotics. However, these effects on phage and bacteria concentration were observed for both phages with and without *acrIF1* and for both CRISPR-immune and CRISPR-KO cells. This suggests that Gm interferes with phage amplification in a way that is independent of CRISPR-Cas and Acr, which results in a lesser impact on bacterial growth. Accordingly, the aminoglycosides antibiotic family, to which Gm belongs, has previously been reported to hinder phage production and favor bacterial growth when used at doses close or above the MIC (24, 36, 37). The cause for the different effects of Gm versus the other three translation inhibitors is not known. This could potentially originate from their inhibitory efficiency, as Chl, Ery, and Tet are bacteriostatic and have a dose-dependent effect on cell growth, whereas Gm is bactericidal and thus mainly affects cell survival above the MIC (42). Moreover, the four antibiotics have different mode of actions, with Chl, Ery, and Tet inhibiting peptide-bond formation (30), while Gm promotes error-prone translation (43).

Owing to the sharp rise in infections by antibiotic-resistant bacterial strains and the health burden that they impose (44), phage treatment is once again seen as a future replacement for chemical antibacterial treatments (45). However, bacteria often evolve resistance to phages quickly and effectively through a range of resistance mechanisms, thereby obliterating any therapeutic effect (2, 3, 46). Combining phage and antibiotics therapy has been proposed as a way to circumvent this, by imposing two different selective pressures on bacteria (47). Such an approach has been studied in in vitro models, and is now being applied in clinical trials, showing promising effects in comparison with phage or antibiotic treatments alone (48, 49) and references herein). Another way to tackle bacterial resistance to phage is the use of natural or engineered phage carrying counter-defense mechanisms. Although this strategy has not yet been tested in clinical use, Acr-phage is now envisioned as a potential phage therapy tool to target CRISPR-immune bacteria (50–53) and have shown promising results in animal models (54). However, we show here evidence of a negative interaction between some antibiotics and Acr-phages, if the targeted bacterium is CRISPR-immune to the phage used for therapy. These results, along with previous work showing antagonism between phage and antibiotic treatment (25) and references herein), highlight the need to test each phage–antibiotic combination as well as the individual treatments, to evaluate potential synergy or antagonism between them. In addition, this negative interaction between translation inhibitor antibiotics and phages might also disrupt phage–host interactions in non-targeted bacterial species. Given the suggested role of phage in structuring and stabilizing microbial communities, such as the gut microbiota (55, 56), disturbing the phage–bacteria interaction network through the use of translation inhibitor antibiotics could therefore have important downstream consequences for human or animal health.

Both biotic and abiotic complexities are known to impact phage–host interactions and their coevolution (57), and the experimental setting used here is necessarily simpler than a natural environment or clinical setting. In this study, we used antibiotic doses near or below the MIC for *P. aeruginosa*. While antibiotics are usually used in high-enough doses to cause lethality, the effective concentration can considerably vary between body compartments, potentially reaching sublethal concentrations (58–61). The extent of in vivo antagonism between Acr-phage and translation inhibitor antibiotics during phage therapy would then depend on the antibiotic used, its MIC in the targeted strain, and pharmacokinetic and pharmacodynamic characteristics of the antibiotic. Overall, the model system used in this study may show differences with in vivo settings in for example the effective antibiotic dose that will need to be further examined before extrapolation to clinical settings.

Previous results, focusing on antibiotic effects on the outcome of the battle between phage and hosts carrying CRISPR-Cas immunity, showed that bacteriostatic antibiotics tip the balance in favor of bacteria by slowing down bacterial and phage replication, and hence allowing more time for bacteria to acquire spacers against invading phages (21). We report here another negative effect on phage by Chl, Ery, and Tet, which are also bacteriostatic antibiotics. This suggests that their impact on phage infectivity could be twofold, by first favoring the acquisition of spacers against phages (as bacteriostatic antibiotics) and then by decreasing the efficiency of phage counter-defense against bacterial CRISPR-Cas system (as translation inhibitors).

## Materials and Methods

### Bacterial and Viral Strains.

The strain derived from UCBPP-PA14 (PA14) of *P. aeruginosa* carrying two spacers targeting the phage DMS3*vir* (CRISPR-immune) and the strain UCBPP-PA14 *csy3::LacZ* (CRISPR-KO) with a non-functional CRISPR system was described in ref. 19. Bacteria were cultured at 37 °C with 180 rpm shaking in Lysogeny Broth (LB) or M9 minimal medium (22 mM Na2HPO4; 22 mM KH2PO4; 8.6 mM NaCl; 20 mM NH4Cl; 1 mM MgSO4; 0.1 mM CaCl2) supplemented with 0.2% glucose (M9 + glucose).

### Phages.

Recombinant lytic phages DMS3*mvir* [derived from phage DMS3*vir* to be targeted by 1 spacer in PA14 and 3 spacers in the CRISPR-immune strain (8)] and DMS3*mvir*-AcrIF1 [expressing Acr protein that blocks the PA14 CRISPR I-F system (34)] were used throughout this study. Phage stocks were obtained from lysates prepared on PA14 CRISPR-KO and stored at 4 °C.

### Cas Expression Assay.

LacZ protein expression was determined as previously described (21) by measuring LacZ activity of PA14 *csy::lacZ* (CRISPR-KO). LacZ activity was measured through the degradation of the b-Galactosidase fluorogenic substrate 4-methylumbelliferyl b-D-galactoside (MUG). An overnight culture of PA14 CRISPR KO grown in LB was diluted to 2 * 10^7^ cfus/mL in 6 mL fresh media in a glass vial, in the presence or absence of antibiotics sub-MIC concentrations (*SI Appendix,* Table S1). Each treatment was performed in 12 independent biological replicates. After 5 h of incubation at 37 °C with 180 rpm shaking, 200 µL of each tube was transferred to a 96-well plate and OD_600_ of was read in a Varioskan Flash Multimode plate reader. The 96-well plate was then frozen at −80 °C for 24 h then defrosted and 10 µL were transferred to a new 96-well plate and frozen again at −80 °C for 1 h. The plate was transferred to 37 °C for 15 min before adding 100 µL of reagent solution (0.25 mg/mL MUG and 2 mg/mL lysozyme in phosphate-buffered saline). Fluorescence (excitation and emission wavelengths at 365 nm and 450 nm, respectively) was measured in a Varioskan Flash Multimode plate reader immediately and 30 min after incubation at 37 °C. Relative fluorescence was calculated as (fluorescence after 30 min—fluorescence after 0 min)/OD_600_.

### Infection Assays in Liquid Medium.

All infections assays were conducted in M9+glucose (22 mM Na2HPO4; 22 mM KH2PO4; 8.6 mM NaCl; 20 mM NH4Cl; 1 mM MgSO4; 0.1 mM CaCl2; 0.2% w/v glucose). Overnight cultures grown in M9+glucose were diluted to 2 * 10^7^ colony-forming units (CFUs)/mL in fresh media. One hundred and eighty microliters of the diluted cells were added to each well of a 96-well plate and were subsequently treated with 10 µL of fresh media containing the appropriate antibiotic concentration (final antibiotic concentration listed in *SI Appendix,* Table S1) and 10 µL of fresh media containing 2*10^7^ PFUs/mL DMS3*mvir* or DMS3*mvir*-AcrIF1 (MOI = 1), or no phage as control. Each treatment was performed in eight independent biological replicates. After 24 h of incubation at 37 °C with 180 rpm shaking, final bacterial concentration was determined by measuring the optical density at l = 600 nm (OD_600_) in a Varioskan Flash Multimode plate reader. Final phage concentration was determined by titration on a soft agar lawn. Phages were extracted by mixing 100 µL of each infection with 10 µL of chloroform. After thorough mixing by pipetting, cells were harvested by spinning at 3,500 rpm for 20 min and the supernatant containing phages was recovered. A mixture of 8 mL of molten LB soft agar (0.5%) and 400 µL of CRISPR-KO cells grown overnight in LB was poured on top of a hard LB agar (1.5%) lawn. Serial dilutions of extracted phages were spotted on this dried soft agar plate and plaques were counted after incubation overnight at 37 °C.

### CRISPR Immunosuppression Experiment.

The CRISPR immunosuppression protocol (Fig. 1) was adapted from ref. 19 as previously described (62). Overnight cultures of PA14 CRISPR-immune or CRISPR-KO grown in LB (approximately 3 * 10^10^ CFUs) were either unexposed or exposed to antibiotic sub-MIC concentrations (*SI Appendix,* Table S1). Subsequently, bacteria were either uninfected or infected with 10^10^ PFUs of DMS3*mvir* or DMS3*mvir*-AcrIF1. Each treatment was performed in six independent biological replicates. After 2 h of incubation at 37 °C with 180 rpm shaking, cells were harvested by spinning at 3,500 rpm for 20 min.

The phage titer was quantified by spotting 4 µL of serially diluted supernatant on LB soft agar plates. After incubation overnight at 37 °C, plaques were counted.

Bacteria pellets were washed twice in 1 mL of 300 mM sucrose and resuspended in 300 µL of 300 mM sucrose. The resuspended bacteria were divided into three 100 µL samples. One sample was serially diluted and plated on LB agar to enumerate total bacterial CFUs before transformation (in order to verify that all treatments have equal bacterial concentration before transformation) and the other two were electroporated with either plasmid pHERD30T (NT) or a pHERD30T-derived plasmid targeted by the PA14 CRISPR-Cas system (targeted, T) (19). Transformed bacteria were allowed to recover for 1 h at 37 °C with 180 rpm shaking in 1 mL of LB. After recovery, bacteria were pelleted and resuspended in 100 µL LB, plated on LB agar plates supplemented with 50 µg/mL Gentamycin to select for transformants, and incubated overnight at 37 °C.

RTE was calculated for each treatment as (number of colonies transformed with targeted plasmid)/(number of colonies transformed with non-targeted plasmid).

### Quantification and Statistical Analysis.

All statistical analyses (two-way ANOVA with Dunnett post hoc test) were done with GraphPad Prism version 9.3.1 and statistical parameters are reported in figure legends or within the *Results* section.

## Supplementary Material

Appendix 01 (PDF)

Dataset S01 (XLSX)

Dataset S02 (XLSX)

Dataset S03 (XLSX)

Dataset S04 (XLSX)

## Data Availability

All study data are included in the article and/or *SI Appendix*.
