# Supplementary material for: Antibiotics that affect translation can antagonize phage infectivity by interfering with the deployment of counter-defenses
Source: Proc Natl Acad Sci U S A. 2023 Jan 20;120(4):e2216084120. doi: 10.1073/pnas.2216084120 (PMC9942909; doi:10.1073/pnas.2216084120)
Supplement: Supplementary file 1 — Appendix 01 (PDF) [file pnas.2216084120.sapp.pdf]

PNAS

Supporting Information for
Antibiotics that affect translation can antagonize phage infectivity by interfering with the deployment of counter-defences.

Benoit J. Pons*, Tatiana Dimitriu, Edze R. Westra, Stineke van Houte*.

*Correspondence: Benoit J. Pons, Stineke van Houte

Email: b.pons@exeter.ac.uk (B.J.P.), c.van-houte@exeter.ac.uk (S.v.H.)

This PDF file includes:

Figure S1
Figure S2
Figure S3
Table S1

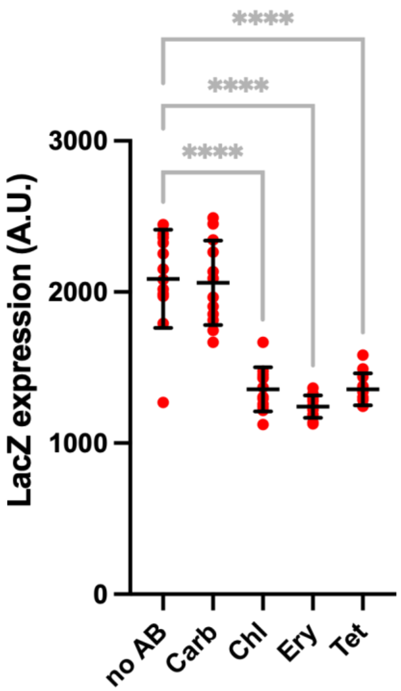

Fig. S1. Translation inhibitor antibiotics inhibit protein production while carbenicillin does not.

Effects of the different antibiotic treatments (see SI Appendix, Table S1) on LacZ activity. Relative fluorescence / OD_{600} is shown for the reporter strain PA14 csy3::lacZ grown for 24h in presence (no AB) or in presence of antibiotic. Each data point represents an independent biological replicate (n = 12), and the mean ± standard deviation for each treatment is displayed as black bars. Asterisks show treatments that are different from the no-antibiotic control (Dunnett, **** p<0.0001).

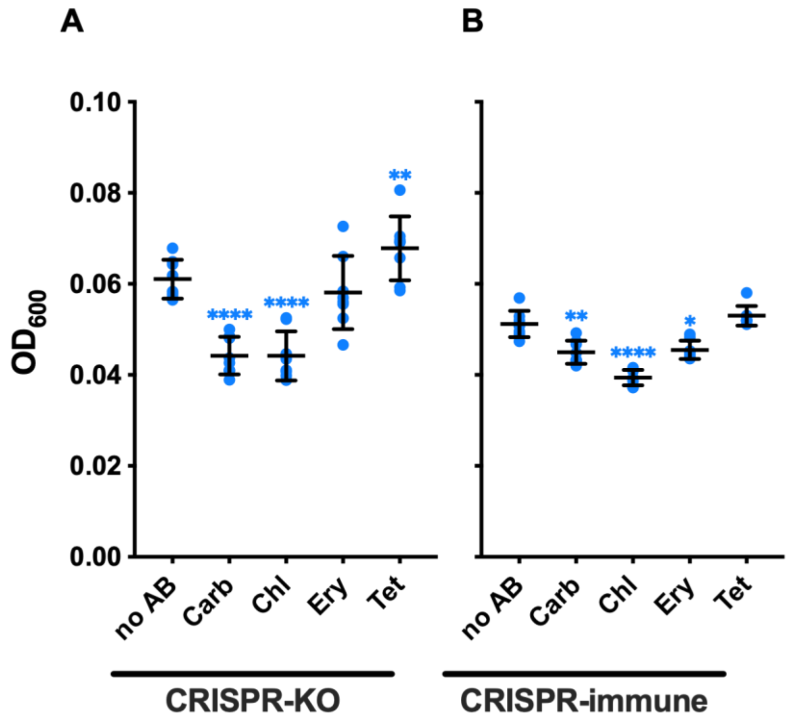

Fig. S2. Translation inhibitor antibiotics do not impact cell growth in the absence of phages.

Effects of the different antibiotic treatments (see SI Appendix, Table S1) on bacterial OD600 of PA14 CRISPR-KO (A) or CRISPR-immune (B) after 24h of growth. Each data point represents an independent biological replicate (n = 8), and the mean ± standard deviation for each treatment is displayed as black bars. Asterisks show treatments that are different from the no-antibiotic control (Dunnett, * $0.01<p<0.05$, ** $0.001<p<0.01$**** $p<0.0001$).

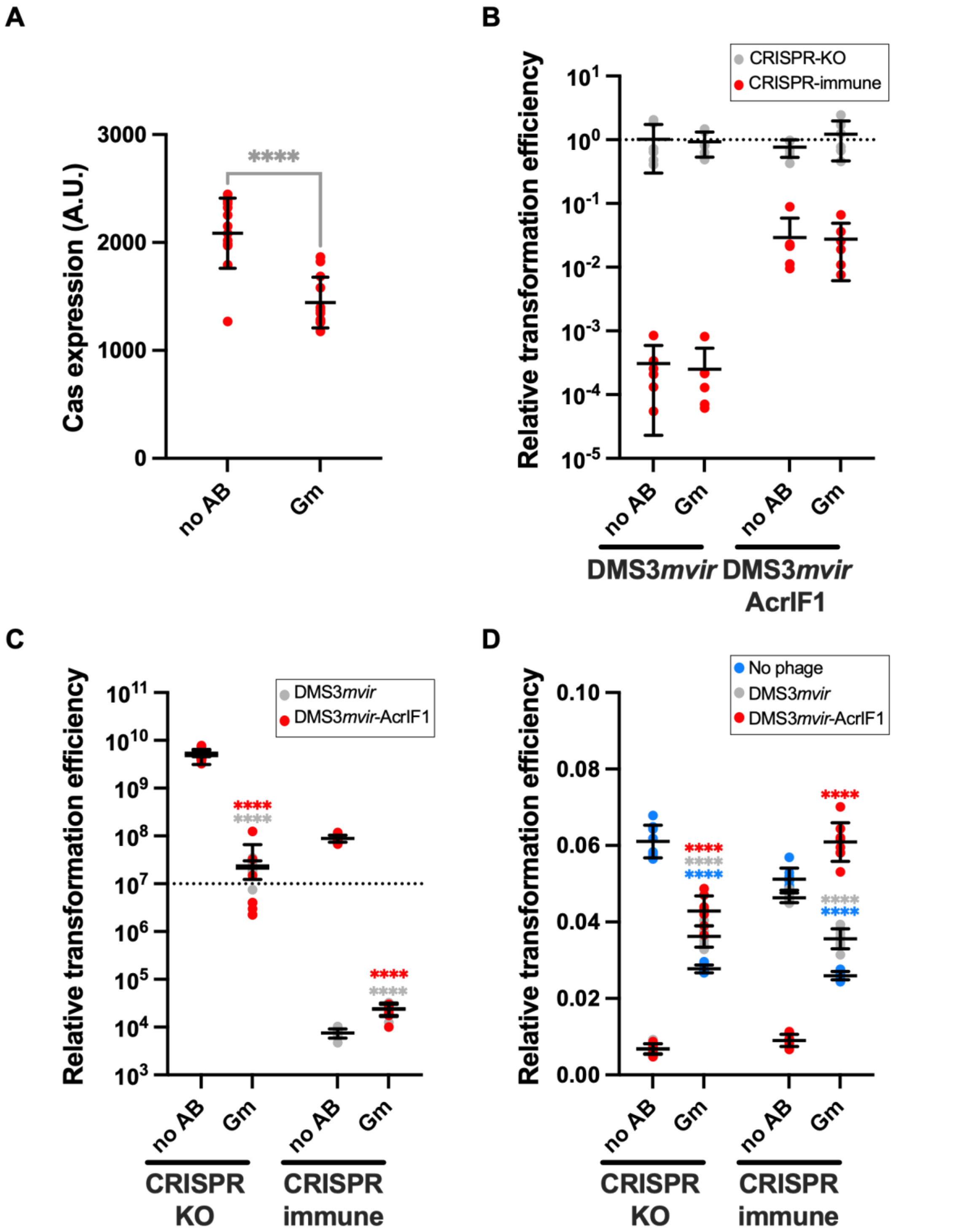

Fig. S3. Gentamycin prevents phage replication in a CRISPR and Acr independent manner.
(A) Effect of gentamycin (see SI Appendix, Table S1) on LacZ activity. Relative fluorescence / OD_{600} is shown for the reporter strain PA14 csy3::lacZ grown for 24h in presence (no AB) or in presence of gentamicin.
(B) Relative transformation efficiencies (targeted plasmid/non-targeted plasmid) of PA14 CRISPR-KO (grey data points) or CRISPR-immune (red data points) pre-infected with phage DMS3*mvir* or DMS3*mvir*-AcrIF1, in the absence (no AB) or presence of gentamycin (see SI Appendix, Table S1).

(C) Effects of gentamycin (see SI Appendix, Table S1) on DMS3*mvir* (grey data points) or DMS3*mvir*-AcrIF1 (red data points) titre after 24h of infection on PA14 CRISPR-KO or CRISPR-immune cells. The dashed line indicates the phage titre at t=0h. The limit of detection is 250 PFUs/mL

(D) Effects of gentamycin (see SI Appendix, Table S1) on bacterial OD_{600} of PA14 CRISPR-KO or CRISPR-immune after 24h of infection by DMS3mvir (grey data points) or DMS3mvir-AcrIF1 (red data points) or in absence of phage (blue data points).

Each data point represents an independent biological replicate (A, n = 12, B, n = 6, C and D, n = 8) and the mean ± standard deviation for each treatment is displayed as black bars. Asterisks show treatments that are different from the no-antibiotic control (Dunnett, **** $p<0.0001$).

Table S1. Antibiotics used in this study.

Antibiotic abbreviations, classes(1), molecular targets(1), overall bacteriostatic/bactericidal(2), minimum inhibitory concentration (MIC, µg/mL)(2) and standard concentrations used (µg/mL) are shown for all antibiotics used in this study.

**Name**		**Class**	**Target**	**Bacteriostatic/ bactericidal**	**MIC (µg/mL)**	**Standard (µg/mL)**
Carbenicillin	Carb	Penicillin	Penicillin binding proteins	bactericidal	25	2.5
Chloramphenicol	Chl	Chloramphenicols	50S ribosome subunits	bacteriostatic	30	25
Erythromycin	Ery	Macrolides	50S ribosome subunits	bacteriostatic	100	100
Tetracycline	Tet	Tetracyclines	30S ribosome subunits	bacteriostatic	10	2.5
Gentamycin	Gm	Aminoglycoside	30S ribosome subunits	bactericidal	1.25	0.63

SI References

1. D. Sohmen, J. M. Harms, F. Schlünzen, D. N. Wilson, SnapShot: Antibiotic Inhibition of Protein Synthesis I. *Cell* **138**, 1248-1248.e1 (2009).

2. T. Dimitriu, *et al.*, Bacteriostatic antibiotics promote CRISPR-Cas adaptive immunity by enabling increased spacer acquisition. *Cell Host Microbe*, S1931312821005205 (2021).
